# Host susceptibility factors render ripe tomato fruit vulnerable to fungal disease despite active immune responses

**DOI:** 10.1093/jxb/eraa601

**Published:** 2021-01-19

**Authors:** Christian J Silva, Casper van den Abeele, Isabel Ortega-Salazar, Victor Papin, Jaclyn A Adaskaveg, Duoduo Wang, Clare L Casteel, Graham B Seymour, Barbara Blanco-Ulate

**Affiliations:** 1 Department of Plant Sciences, University of California, Davis, Davis, CA, USA; 2 Laboratory of Plant Physiology, Wageningen University, Wageningen, The Netherlands; 3 Ecole Nationale Supérieure Agronomique de Toulouse, Toulouse, France; 4 School of Integrative Plant Science, Cornell University, Ithaca, NY, USA; 5 School of Biosciences, Plant and Crop Science Division, University of Nottingham, Sutton Bonington, Loughborough, UK; 6 CONICET – National University of La Plata, Argentina

**Keywords:** *Botrytis cinerea*, fruit–pathogen interactions, fruit ripening, *Fusarium acuminatum*, immune responses, non-ripening mutants, pectate lyase, preformed defenses, *Rhizopus stolonifer*, susceptibility factors

## Abstract

The increased susceptibility of ripe fruit to fungal pathogens poses a substantial threat to crop production and marketability. Here, we coupled transcriptomic analyses with mutant studies to uncover critical processes associated with defense and susceptibility in tomato (*Solanum lycopersicum*) fruit. Using unripe and ripe fruit inoculated with three fungal pathogens, we identified common pathogen responses reliant on chitinases, WRKY transcription factors, and reactive oxygen species detoxification. We established that the magnitude and diversity of defense responses do not significantly impact the interaction outcome, as susceptible ripe fruit mounted a strong immune response to pathogen infection. Then, to distinguish features of ripening that may be responsible for susceptibility, we utilized non-ripening tomato mutants that displayed different susceptibility patterns to fungal infection. Based on transcriptional and hormone profiling, susceptible tomato genotypes had losses in the maintenance of cellular redox homeostasis, while jasmonic acid accumulation and signaling coincided with defense activation in resistant fruit. We identified and validated a susceptibility factor, pectate lyase (*PL*). CRISPR-based knockouts of *PL*, but not polygalacturonase (*PG2a*), reduced susceptibility of ripe fruit by >50%. This study suggests that targeting specific genes that promote susceptibility is a viable strategy to improve the resistance of tomato fruit against fungal disease.

## Teaser

Increased susceptibility to fungal disease during tomato ripening is driven by the accumulation of susceptibility factors, particularly a pectin degrading enzyme.

## Introduction

Half of all fruit and vegetables produced globally are lost each year (Gustavsson *et al.*, 2011). While the causes of losses vary by region and commodity, fungal phytopathogens have a widespread role, as 20–25% of all harvested fruit and vegetables are lost to rotting caused by such fungi (Sharma *et al.*, 2009). In fleshy fruits, this issue is exacerbated because, in general, fruit become more susceptible to fungal pathogens as they ripen (Prusky, 1996; Blanco-Ulate *et al.*, 2016b). Ripening-associated susceptibility has been demonstrated in multiple commodities including climacteric fruits such as tomato, stone fruit, banana, apple, and pear, as well as non-climacteric fruits such as strawberry, cantaloupe, citrus, and pineapple (Zhang *et al.*, 1999; Gell *et al.*, 2008; Morales *et al.*, 2008; Cantu *et al.*, 2009; Lassois *et al.*, 2010; Chiu *et al.*, 2013; Alkan *et al.*, 2015; Barral *et al.*, 2019; Lafuente *et al.*, 2019; Petrasch *et al.*, 2019).

The most devastating postharvest pathogens in fruit are those with necrotrophic lifestyles, which deliberately kill host tissue, resulting in rotting. Example pathogens include the model necrotrophic fungi *Botrytis cinerea* and *Sclerotinia sclerotiorum* as well as *Monilinia* spp., *Alternaria* spp., *Rhizopus* spp., *Penicillium* spp., and *Fusarium* spp. (Nunes, 2012; Bautista-Baños, 2014; van Kan *et al.*, 2014; Liang and Rollins, 2018; Petrasch *et al.*, 2019). Plant immune responses against necrotrophic fungi are multi-layered, involving (i) recognition of pathogen-associated molecular patterns, such as chitin or chitosan, by pattern recognition receptors, (ii) intracellular signaling through mitogen-activated protein (MAP) kinase cascades, (iii) induction of downstream defenses by coordinated activity of phytohormones, particularly ethylene and jasmonic acid (JA), (iv) cell wall fortifications, and (v) production of various secondary metabolites and antifungal proteins (van der Ent and Pieterse, 2012; Mbengue *et al.*, 2016; Pandey *et al.*, 2016; AbuQamar *et al.*, 2017; Veloso and van Kan, 2018). However, most defense strategies have been studied in leaves, and their utilization and effectiveness in fruit have been assessed only with single pathogens (Cantu *et al.*, 2009; Alkan *et al.*, 2015; Ahmadi-Afzadi *et al.*, 2018).

The outcome of any fruit–necrotroph interaction relies on the balance between the presence or induction of defenses and the contributions of susceptibility factors. Though induced defenses are heavily studied in plant immunity, the impact of preformed (or ‘constitutive’) defenses and susceptibility factors are less researched (van Schie and Takken, 2014). Preformed defenses include structural barriers, such as the cell wall and cuticle, and the accumulation of secondary metabolites (Wittstock and Gershenzon, 2002; Veronese *et al.*, 2003), while susceptibility factors include the abundance of simple sugars and organic acids or activity of host cell wall modifying proteins (Cantu *et al.*, 2008; Centeno *et al.*, 2011). A sufficient understanding of ripening-associated susceptibility requires a characterization of the ripening program’s impact on (i) the ability of the host to express necessary defense genes upon pathogen challenge, (ii) the integrity of preformed defenses, and (iii) the abundance of susceptibility factors.

In this study, we first applied a transcriptomic approach to characterize core tomato fruit responses to three fungal pathogens and changes in gene expression that occur during ripening to promote susceptibility. To identify core responses that are not merely pathogen-specific, we used three pathogens with necrotrophic infection strategies: *B. cinerea*, *Rhizopus stolonifer*, and *Fusarium acuminatum*. Using well-established defense gene classifications, we developed profiles of host defense gene expression responses in unripe and ripe fruit. We then determined the susceptibility phenotypes of three non-ripening mutants: *Colorless non-ripening* (*Cnr*), *ripening inhibitor* (*rin*), and *non-ripening* (*nor*), which have unique defects in ripening features (Vrebalov *et al.*, 2002; Giovannoni *et al.*, 2004; Manning *et al.*, 2006; Ito *et al.*, 2017; Gao *et al*., 2019, 2020; Wang *et al.*, 2019b). After demonstrating that each mutant has distinct susceptibility to disease, we identified ripening genes whose expression changes may impact the disease outcome. By integrating our transcriptomic data and mutant analyses, we found preformed defenses and susceptibility factor candidates associated with *B. cinerea* infections. Using CRISPR-based mutants, we established that one candidate, the pectin-degrading enzyme pectate lyase, is indeed a disease susceptibility factor in ripe tomato fruit.

## Materials and methods

### Plant material

Tomato (*Solanum lycopersicum*) cv. ‘Ailsa Craig’ (AC), isogenic non-ripening mutants *rin*, *nor*, and *Cnr*, and CRISPR-based *PL* (PL5-4) and *PG2a* (PG21) mutants with azygous control plants (Wang *et al.*, 2019a) were grown under standard field conditions in the Department of Plant Sciences Field Facilities at the University of California, Davis. Fruit were tagged at 3 d post-anthesis (dpa) and harvested at 31 dpa for mature green (MG) and at 42 dpa for red ripe (RR) or equivalent for ripening mutants. For all tomato genotypes, fruit ripening stages were visually assessed based on color, and quality attributes were measured at the time of harvest (see Supplementary Tables S1, S2). Color was also assessed quantitatively using a Minolta CR-400 chroma meter (Konica Minolta Sensing Inc., Japan) and recorded in the L*a*b color space for the non-ripening fruit (*n*=24–48 fruit). Firmness was evaluated with a TA.XT2i Texture Analyzer (Texture Technologies, USA) using a TA-25 cylinder probe, a trigger force of 0.045 N and a test speed of 2.00 mm s^−1^. Non-ripening fruit (*n*=20–25 fruit) were evaluated at both stages, azygous and CRISPR lines (*n*=32 fruit) only at the RR stage. Soluble solids, titratable acidity (TA) levels, and pH were determined from the juice of the same fruit used for firmness measurements (*n*=4–9 replicates of a pool of 5–8 fruit each). Soluble solids were measured as degrees Brix with a Reichert AR6 Series automatic bench refractometer (Reichert Inc., USA). TA and pH were measured with the TIM850 Titration Manager (Radiometer Analytics, Germany). Four grams of juice diluted in 20 ml deionized water were titrated to determine TA based on citric acid equivalents. Significant differences in physiological parameters between genotypes were determined with analysis of variance (ANOVA) followed by *post hoc* testing (Tukey’s honestly significant difference, HSD) using R (R Foundation for Statistical Computing, Vienna, Austria).

### Fungal culture and fruit inoculation

*Rhizopus stolonifer* and *F. acuminatum* isolates were taken from rotting fruit and identified through morphological and sequencing methods (Petrasch *et al.*, 2019). *Botrytis cinerea* (B05.10), *R. stolonifer*, and *F. acuminatum* cultures were grown on 1% potato dextrose agar media. Conidia were harvested from sporulating cultures in 0.01% Tween-20 (Sigma-Aldrich, USA) and counted. Fruit were disinfected and inoculated as described in Petrasch *et al.*, 2019 using 500, 30, and 1000 conidia μl^−1^ for *B. cinerea*, *R. stolonifer*, and *F. acuminatum*, respectively. Each fruit used to measure disease incidence and severity was punctured at six sites; each fruit used for RNA extraction and transcriptomic analysis was punctured at 15 sites. No inoculum was introduced at puncture sites on wounded fruit. Healthy controls were not wounded or inoculated. Fruit were incubated for up to 3 d at 25 °C in high-humidity containers (90% relative humidity). Five replicates (*n*=8–10 fruit each) of each treatment were generated for transcriptomic analysis, while four replicates (*n*=10–12 fruit each) were used for measurements of disease progression.

### Disease incidence and severity measurements

Fruit disease incidence and severity were measured at 1, 2, and 3 d post-inoculation (dpi). Disease incidence was the percentage of inoculated sites displaying visual signs of tissue maceration or soft rot. Disease severity was calculated as the average lesion diameter (in mm) of each inoculated site displaying signs of rot. Significant differences in disease incidence and severity between genotypes were assessed for each pathogen with ANOVA followed by Tukey’s HSD using R.

### RNA extraction and library preparation

At 1 dpi, fruit pericarp and epidermal tissue of the blossom end halves of healthy, wounded, and infected fruit were collected and immediately frozen in liquid nitrogen and lysed using a Retsch Mixer Mill MM 400 (Retsch, Germany). RNA was extracted from 1 g of ground material as described in Blanco-Ulate *et al.* (2013). The purity and concentration of the extracted RNA were determined with a NanoDrop One Spectrophotometer (Thermo Fisher Scientific, USA) and a precise concentration measurement was made with a Qubit 3 fluorometer (Thermo Fisher Scientific). The integrity of the RNA was confirmed by agarose gel electrophoresis.

One hundred and twenty-six cDNA libraries were prepared using the Illumina TruSeq RNA Sample Preparation Kit v.2 (Illumina, USA) from isolated RNA (*n*=3–8 libraries per treatment). Each library was barcoded and analysed with the High Sensitivity DNA Analysis Kit for the Agilent 2100 Bioanalyzer (Agilent Technologies, USA). Libraries were sequenced as single-end 50-bp reads on an Illumina HiSeq 4000 platform by the DNA Technologies Core at the UC Davis Genome Center.

### RNA sequencing and data processing

Raw sequencing reads were trimmed for quality and adapter sequences using Trimmomatic v0.33 (Bolger *et al.*, 2014) with the following parameters: maximum seed mismatches=2, palindrome clip threshold=30, simple clip threshold=10, minimum leading quality=3, minimum trailing quality=3, window size=4, required quality=15, and minimum length=36. Trimmed reads were mapped using Bowtie2 (Langmead and Salzberg, 2012) to combined transcriptomes of tomato (SL4.0 release; http://solgenomics.net) and one of the three pathogens: *B. cinerea* (http://fungi.ensembl.org/Botrytis_cinerea/Info/Index), *F. acuminatum* (Petrasch *et al.*, 2019), or *R. stolonifer* (Petrasch *et al.*, 2019). Count matrices were made from the Bowtie2 results using sam2counts.py v0.91 (https://github.com/vsbuffalo/sam2counts/). Only reads that mapped to the tomato transcriptome were used in the following analyses. A summary of the read mapping results can be found in Supplementary Table S3.

### Differential expression analysis

The Bioconductor package DESeq2 (Love *et al.*, 2014) was used to perform normalization of read counts and differential expression analyses for various treatment comparisons. Differentially expressed (DE) genes for each comparison were those with an adjusted *P*-value of ≤0.05.

### Functional annotation and enrichment analyses

Gene Ontology (GO) terms were retrieved from SolGenomics. Annotations for transcription factors and kinases were generated using the automatic annotation tool from iTAK (Zheng *et al.*, 2016). NBS-LRR family members were identified from Andolfo *et al.*, 2014. Kyoto Encyclopedia of Genes and Genomes (KEGG) annotations were determined using the KEGG Automatic Annotation Server (Moriya *et al.*, 2007), and hormone annotations were derived from these (see Supplementary Table S4). GO enrichments were performed with the goseq package in R (Young *et al.*, 2010), while enrichments for all other annotations were performed using Fisher’s test with resulting *P*-values adjusted via the Benjamini–Hochberg method (Benjamini and Hochberg, 1995).

### Measurement of phytohormones

Ethylene emission was measured in MG and RR fruit (*n*=4 replicates of a pool of 8–10 fruit each) from the day of harvest through 3 dpi. Headspace gas (3 ml) from weighed fruit in sealed 1 liter containers was extracted after 30–90 min in a Shimadzu CG-8A gas chromatograph (Shimadzu Scientific Instruments, Kyoto, Japan). Sample peaks were measured against an ethylene standard of 1 ppm. Ethylene production was calculated from the peak height, fruit mass, and incubation time. JA was measured using liquid chromatography coupled to tandem mass spectrometry and internal standards as in Patton *et al.* (2020) with modifications (*n*=4 replicates of a pool of 8–10 fruit each). Briefly, frozen tissue was lyophilized, weighed and extracted in isopropanol: H_2_O: HCL_1mol_ (2:1:0.005) with 100 μl of internal standard solution (1000 pg) as previously described (Casteel *et al.*, 2015). Samples were evaporated to dryness, resuspended in 100 μl of MeOH, filtered, and 10 µl samples injected into an Agilent 6420 Triple Quad Mass Spectrometer (Agilent Technologies, USA). A Zorbax Extend-C18 column 3.0×150 mm (Agilent) with 0.1% formic acid in water (A) and 0.1% (v/v) formic acid in acetonitrile (B) at a flow rate of 600 ml min^–1^ was used. The gradient was 0–1 min, 20% B; 1–10 min, linear gradient to 100% B; 10–13 min, 100% A. Differences in hormone levels among treatments, ripening stages, and time points were assessed by ANOVA followed by Tukey’s HSD using R.

## Results

### Susceptibility of tomato fruit to fungal infections by *Botrytis cinerea*, *Fusarium acuminatum*, and *Rhizopus stolonifer* increases during ripening

**Fig. 1.**
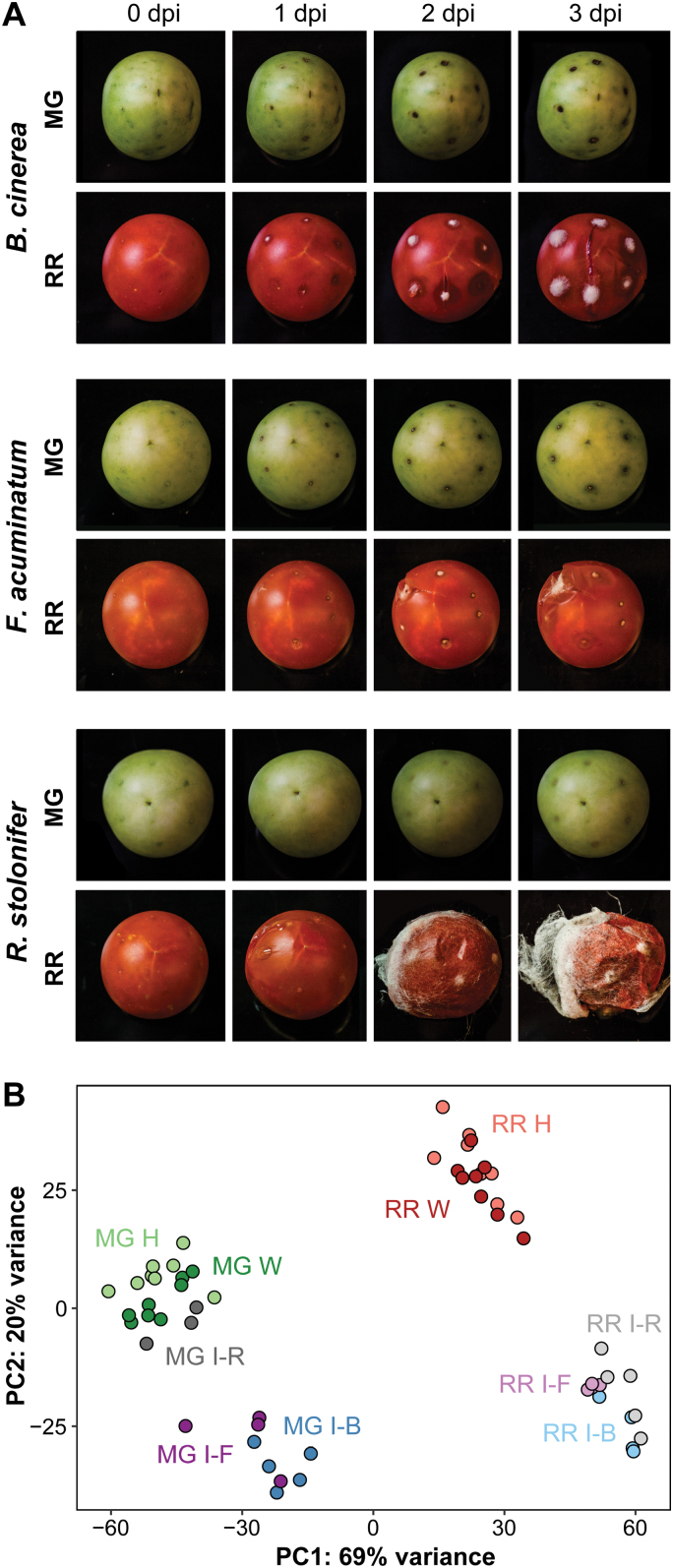
Tomato fruit responses to *B. cinerea*, *F. acuminatum*, and *R. stolonifer*. (A) Disease progression in inoculated mature green (MG) and red ripe (RR) fruit each day up to 3 d post-inoculation (dpi). (B) Principal component analysis of total mapped RNA-Seq tomato reads. Color corresponds to treatment. H, healthy; I, inoculated 1 dpi; W, wounded; B, *B. cinerea*; F, *F. acuminatum*; R, *R. stolonifer*.

To characterize tomato fruit responses to fungal infection at unripe (MG) and ripe (RR) stages, we inoculated fruit (cv. ‘Ailsa Craig’) with *B. cinerea*, *F. acuminatum*, or *R. stolonifer* spores. Each pathogen successfully infected RR fruit, producing visible water-soaked lesions and mycelial growth by 3 dpi, whereas MG fruit remained resistant and, except in samples inoculated with *R. stolonifer*, had a dark, necrotic ring around the inoculation sites (Fig. 1A), a feature of the pathogen response that did not appear in wounded fruit. Thus, MG fruit resistance and RR fruit susceptibility are a feature common to multiple necrotrophic infections. We hypothesized that these susceptibility phenotypes are the result of (i) differences in immune responses at each ripening stage and (ii) developmental processes during ripening that alter the levels of preformed defenses and susceptibility factors. First, we assumed that, compared with a robust immune response in MG fruit, RR fruit have a weaker response, consisting of fewer genes induced, less diverse functionality, and absent expression of critical genes. Additionally, we predicted that ripening may decrease the expression of preformed defenses and increase the expression of susceptibility factors, which create a more favorable environment for infection.

### Susceptible ripe fruit respond to pathogens with a larger, more diverse set of defense genes than resistant unripe fruit

To test if immune responses to fungal pathogens are compromised in RR compared with MG fruit, we sequenced mRNA from *B. cinerea-*, *F. acuminatum-*, and *R. stolonifer-*inoculated fruit at 1 dpi, an early time point at which either a resistant or a susceptible phenotype becomes apparent. We included healthy and wounded MG and RR fruit from the same time point as controls. A principal component analysis (PCA) of the mapped normalized reads for all tomato genes (Fig. 1B) revealed that the major driver separating sample data was the ripening stage (PC1, 69%), while inoculation status accounted for less of the separation (PC2, 20%). The one exception to this pattern was the *R. stolonifer*-inoculated MG samples, which clustered with the healthy and wounded MG samples, suggesting that unripe fruit did not display strong responses to this pathogen and yet remained resistant. However, quantification of normalized pathogen reads (see Supplementary Fig. S1A) confirmed that all three pathogens were detectable at 1 dpi even in MG samples.

**Fig. 2.**
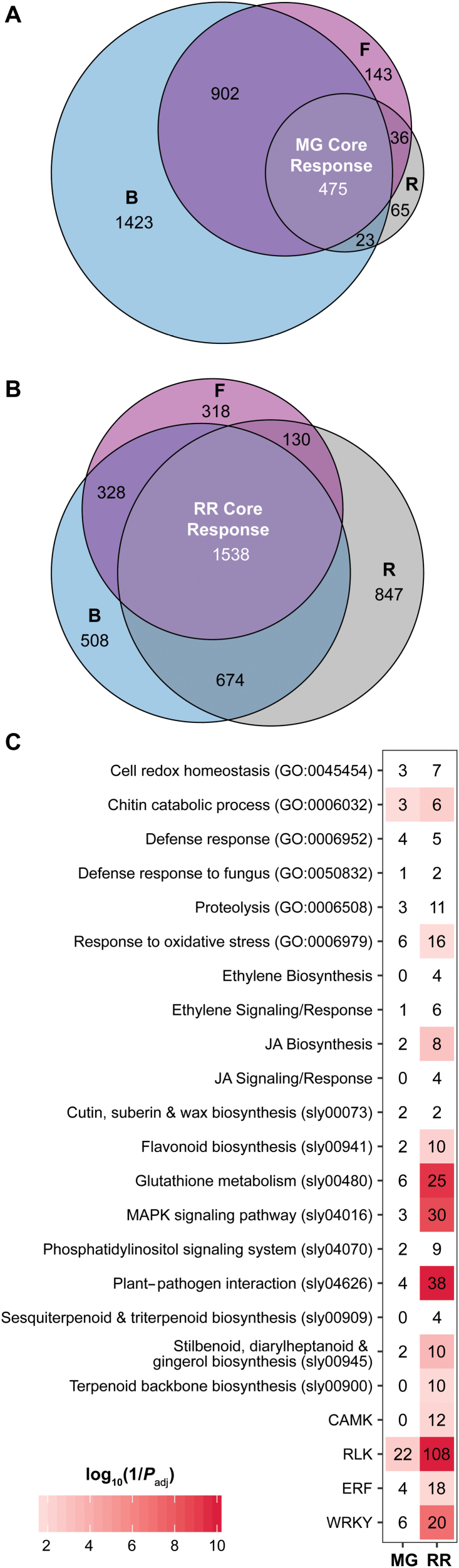
Tomato core responses to fungal inoculations. (A, B) Euler diagram of tomato genes up-regulated in response to inoculation in mature green (MG) (A) or red ripe (RR) (B) fruit. B, *B. cinerea*; F, *F. acuminatum*; R, *R. stolonifer*. Core responses are shown in white. (C) Enrichments of various defense-related classes in the MG and RR core responses. The scale is the log_10_(1/*P*_adj_). Values greater than 10 were converted to 10 for scaling purposes. Numbers in each tile indicate the number of genes within each classification. CAMK, calmodulin-dependent protein kinase; ERF, ethylene responsive factor; JA, jasmonic acid; MAPK, mitogen-activated protein kinase; RLK, receptor-like kinase.

To identify the responses for each ripening stage common to all three pathogens, we performed a differential expression analysis between inoculated and healthy samples for MG and RR fruit. We chose the healthy samples as controls for these comparisons in order to capture responses to necrotrophic infection, which may share features with mechanical wounding. Of all 34 075 protein-coding genes found in the tomato transcriptome, 9366 (27.5%) were found to be differentially expressed (*P*_adj_<0.05) in response to inoculation in fruit at 1 dpi in at least one comparison (see Supplementary Table S4). Of these, 475 genes were significantly up-regulated in MG fruit in response to all three pathogens, corresponding to the MG core response (Fig. 2A), whereas 1538 genes formed the RR core response (Fig. 2B). The MG core response overlapped substantially with the wounding response in MG fruit (Supplementary Fig. S1B), which suggests that unripe fruit activate similar functions when responding to pathogen attack and mechanical damage. However, this large overlap is also due to the similarity between the gene expression profiles of wounded and *R. stolonifer*-inoculated samples as seen in the PCA (Fig. 1B). In contrast, the lack of a strong wounding response in RR fruit indicates that nearly all RR core response genes were strictly pathogen-related (Supplementary Fig. S1B). Down-regulated genes in response to infection were largely unique to each pathogen, with only 57 and 225 down-regulated across all three pathogens in MG and RR fruit, respectively, and thus we decided to continue our analysis only on the up-regulated core response genes. Complete lists of gene set intersections of up-regulated and down-regulated genes are in given in Supplementary Table S5.

We then assessed the MG and RR core responses for the presence of various well-established gene classifications related to pathogen defense, including selected GO terms, KEGG pathways, transcription factor (TF) families, hormone biosynthesis, signaling and response genes, and receptor-like kinase (RLK) genes (Fig. 2C). For each category, we performed enrichment analyses (*P*_adj_<0.05) to identify classifications of particular importance in both MG and RR core responses. A total of 70 defense genes were identified in the MG core response. Interestingly, these were enriched in only two categories: chitin catabolic process (GO:0006032) and RLK genes. The RR core response was enriched in 13 defense categories, including the plant–pathogen interaction (sly04626) and MAP kinase signaling pathways (sly4016), secondary metabolite biosynthesis pathways (sly00900, sly00941, sly00945), WRKY and ethylene responsive factor (ERF) transcription factors, RLKs, and JA biosynthesis. Altogether, 302 defense genes were identified among the RR core response. Thus, in contrast to their respective susceptibility phenotypes, RR fruit appear to mount a more robust and diverse immune response than MG fruit early during inoculation, demonstrating that, contrary to our initial hypothesis, weakened immune responses in RR fruit are not a contributor to ripening-associated susceptibility.

**Fig. 3.**
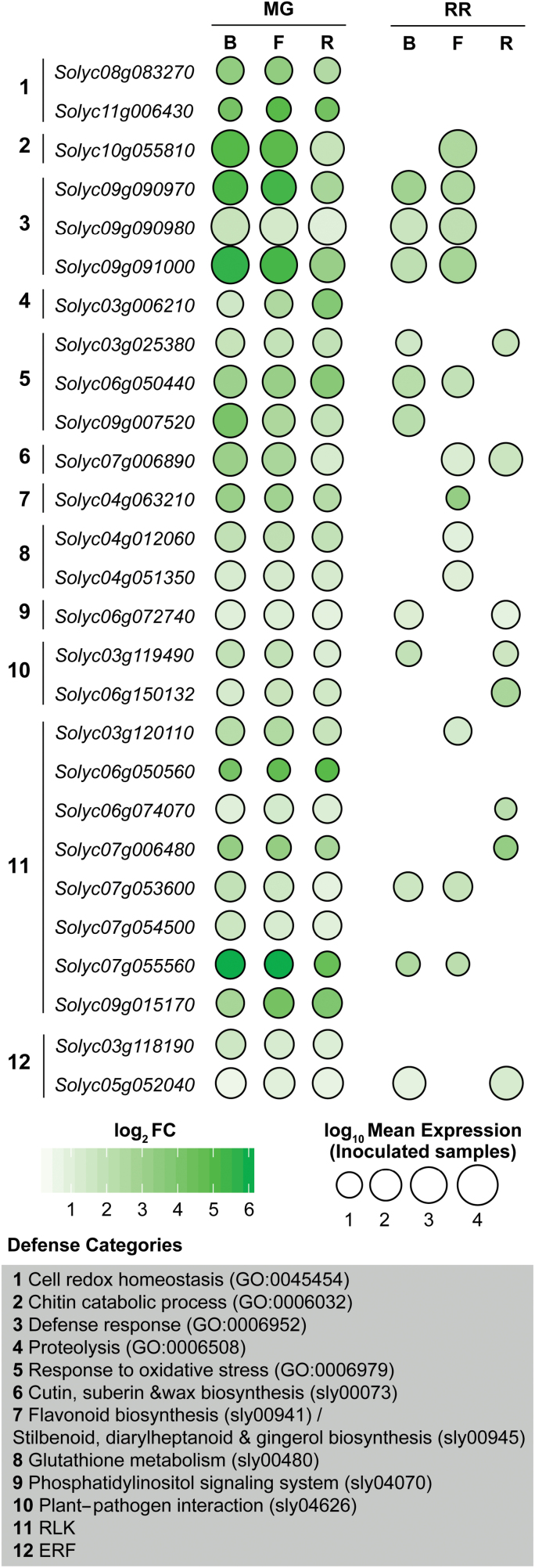
Defense genes in the mature green (MG) core response that are not in the red ripe (RR) core response. Circle sizes are proportional to the average normalized read count values from the inoculated fruit samples. B, *B. cinerea*; F, *F. acuminatum*; R, *R. stolonifer*; ERF, ethylene responsive factor; RLK, receptor-like kinase.

However, it is possible that tomato fruit resistance to necrotrophs could be determined by a small number of genes that were exclusive to the MG core response. Out of the 70 defense genes in the MG core response, 27 were not found in the RR core response (Fig. 3). These 27 genes are heterogeneous, representing 12 different defense categories. Notable genes in this category include a three-gene cluster of PR-10 family proteins (GO:0006952), a chitinase previously identified during infections of tomato with *Cladiosporum fulvum* (*Solyc10g055810*, Danhash *et al.*, 1993), and an ERF active at the onset of ripening (Liu *et al.*, 2015a). Although these 27 genes were not in the RR core response, most of them were induced during RR infections by one or two of the pathogens studied. Only seven were not up-regulated by any of the three pathogens in RR fruit, including the ERF mentioned above (*Solyc03g118190*), as well as three RLK genes, two glutaredoxin genes involved in the response to oxidative stress, and a cysteine protease. Given that each of these genes belongs to a large family of genes whose members are often functionally redundant, and their average expression levels in infected MG fruit were fairly low (normalized read counts 8.13–149.07), we consider it unlikely that the lack of these genes in the RR core response contributes heavily to susceptibility.

Additionally, the induction of defense genes in the RR core response could be ineffective if their expression levels were too weak compared with those seen in resistant MG fruit. We evaluated the levels of gene expression in inoculated RR fruit via a differential expression comparison (*P*_adj_<0.05) to inoculated MG fruit. Of all the RR core defense genes identified above, 269/302 (89.1%) were expressed at equal or greater levels (average log_2_FC=2.16) in inoculated RR fruit compared with inoculated MG fruit for all three pathogens. Conversely, 33/302 (11.9%) of these defense genes were expressed at higher levels in MG fruit compared with RR fruit for at least one of the three pathogens (see Supplementary Table S6). These genes are diverse, representing 15 different defense categories. Prominent genes in this category include *TAP1* (*Solyc02g079500*) and *TAP2* (*Solyc02g079510*), two peroxidases associated with defensive suberization in tomato (Roberts and Kolattukudy, 1989; Kesanakurti *et al.*, 2012); *CHI3* (*Solyc02g082920*) and *CHI17* (*Solyc02g082930*), two chitinases associated with *C. fulvum* infection (Danhash *et al.*, 1993); and the JA biosynthesis gene *OPR3* (*Solyc07g007870*). While it is possible that resistance may be determined by these genes, these results indicated that the differences in immune responses observed between MG and RR fruit are not likely solely responsible for differences in susceptibility, and, therefore, we considered the alternate hypothesis.

### Defects in the regulation of ripening indicate that only some ripening processes promote susceptibility to fungal disease

We explored the possibility that the increase in susceptibility to fungal pathogens is heavily influenced by a decline of preformed defenses and accumulation of susceptibility factors that occur during fruit ripening prior to pathogen challenge. Due to the complexity of the ripening program, we utilized isogenic non-ripening tomato mutants as tools to identify specific developmental features that are integral to fruit resistance or susceptibility. The *Cnr*, *rin*, and *nor* mutants produce fruit that lack most of the characteristic changes associated with normal ripening, such as color, texture, acidity, sugar accumulation, and ethylene production, but yet are phenotypically different from one another (see Supplementary Table S1). All three mutant lines likely result from spontaneous gain-of-function mutations in transcription factors with key roles in the regulation of ripening (Ito *et al.*, 2017; Gao *et al*., 2019, 2020; Wang *et al.*, 2019b).

**Fig. 4.**
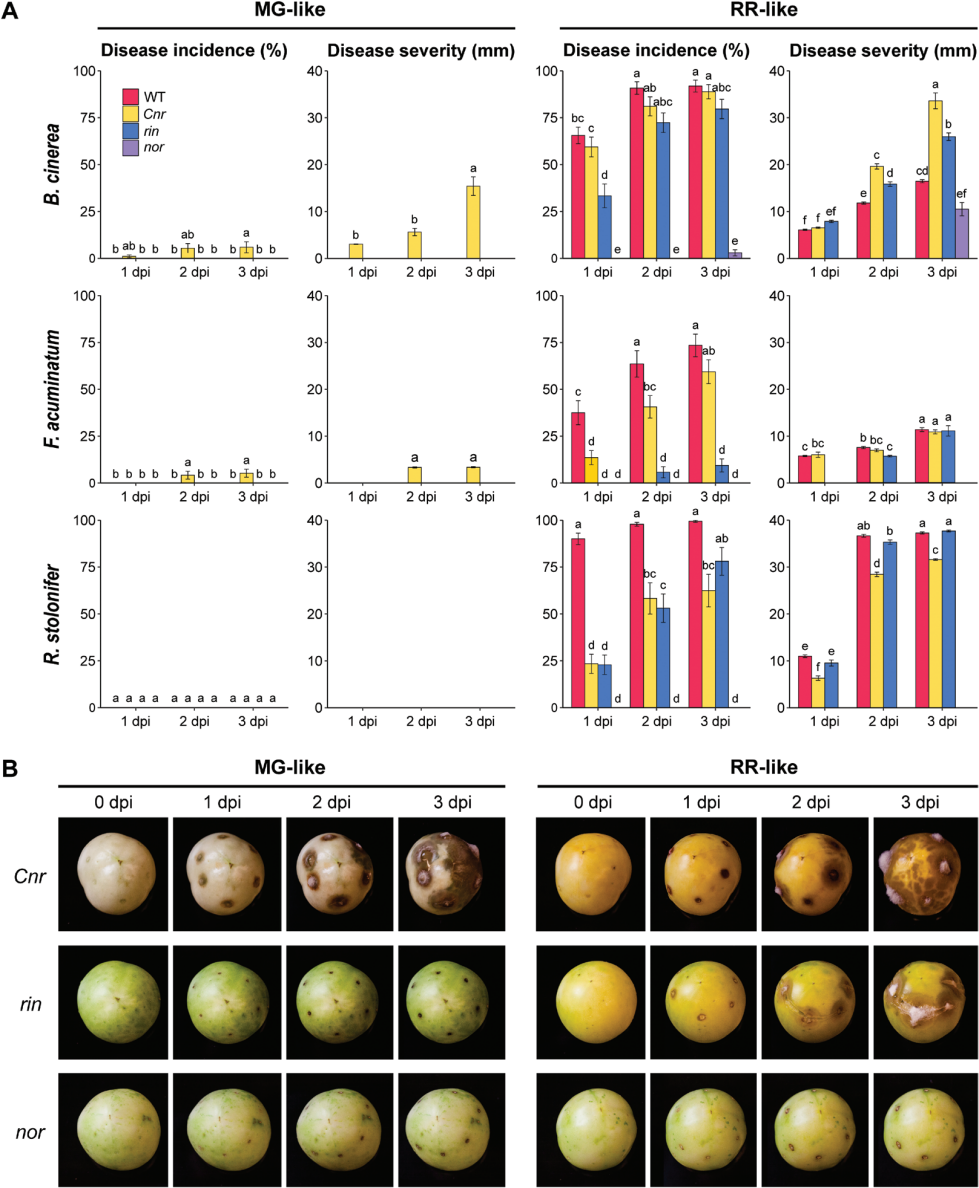
Susceptibility of the non-ripening mutants *Cnr*, *rin*, and *nor* to fungal infections. (A) Disease incidence and severity measurements for MG-like (left) and RR-like (right) fruit. Wild-type values are included for comparison. (B) Disease progression of *B. cinerea*-inoculated MG-like and RR-like fruit each day up to 3 d post-inoculation. Letters represent statistical differences between genotypes for each pathogen in study (*P*<0.05).

We inoculated fruit of these mutant genotypes at comparable stages to MG and RR wild-type fruit (i.e. ‘MG-like’ and ‘RR-like’) with *B. cinerea*, *F. acuminatum*, and *R. stolonifer* and measured disease incidence and severity up to 3 dpi (Fig. 4). For all three pathogens at both MG-like and RR-like stages, only *nor* fruit were consistently resistant to infection. MG-like fruit of *Cnr* were the only unripe fruit susceptible to any pathogen, with both *B. cinerea* and *F. acuminatum* able to produce lesions on a significant number of these fruit. Consistent with this, *Cnr* RR-like were more susceptible than wild-type RR fruit to *B. cinerea*, with average disease severity (i.e. lesion size) nearly twice as great at 3 dpi (Fig. 4A). The fruit of *rin* at both MG-like and RR-like stages showed similar or slightly lower susceptibility to all pathogens when compared with wild-type, with the exception of a significant reduction in disease incidence to *F. acuminatum* at the RR-like stage. Because some ripening processes may promote susceptibility, others may maintain resistance, and others may have no impact, we hypothesized that the *Cnr*, *rin*, and *nor* mutations differentially affect ripening-associated genes or pathways that are critical to tip the balance towards either susceptibility or resistance.

We sequenced mRNA from *B. cinerea*-inoculated and healthy fruit from the non-ripening mutants at MG-like and RR-like stages at 1 dpi. We chose *B. cinerea* inoculations because this pathogen showed the clearest differences in susceptibility phenotypes between these genotypes. We first characterized transcriptional responses of mutant fruit to pathogen challenge by using enrichment analysis of defense-related processes to determine if differences in immune responses could explain the distinct susceptibility phenotypes (Supplementary Fig. S2A). In most cases, the mutant fruit exhibited similar patterns of defense classification enrichments as wild-type fruit in both stages, with some notable exceptions. Compared with the other genotype–stage combinations, *Cnr* MG-like responses were deficient (i.e. less enriched) in the expression of genes from several prominent defense classifications, including chitin catabolic process (GO:0006032), the plant–pathogen interaction (sly04626) and glutathione metabolism (sly00480) pathways, ERF and WRKY transcription factors, and RLK and CAMK genes. Given that *Cnr* fruit were the only genotype at the MG-like stage to display susceptibility to *B. cinerea* infection, it can be suggested that these defense processes may be necessary for resistance in unripe fruit. However, these processes were enriched in the susceptible RR-like fruit of *Cnr* and *rin*, as well as wild-type RR fruit, which clearly indicates that they are not sufficient to result in a resistant outcome.

The role of ethylene and JA showed some variation amongst the mutants. For example, the responses of resistant *nor* fruit in both MG-like and RR-like fruit were noticeably less enriched in ethylene-associated pathways and more enriched in JA-associated pathways. These results suggest that JA-mediated defenses may contribute to tomato fruit resistance in the absence of ethylene, and that the *nor* mutation may activate JA-associated resistance. In support of this observation, levels of JA in healthy fruit appeared to be linked to resistance: they were highest in RR-like *nor* fruit, and only *nor* fruit experienced an increase in JA in the transition from MG-like to RR-like (see Supplementary Table S7). Ethylene levels increase dramatically during ripening in wild-type fruit, but they remain low in all three non-ripening mutants (Supplementary Fig. S2B). However, both *Cnr* and *rin* mutants produce ethylene in response to *B. cinerea* inoculation, with ethylene production in inoculated *Cnr* MG-like fruit reaching levels nearly three times greater than wild-type MG fruit by 3 dpi. Moreover, ethylene signaling/response genes are highly enriched in *Cnr* MG-like fruit responses (Supplementary Fig. S2A). In contrast, healthy *nor* fruit did not produce substantial amounts of ethylene at either stage, and inoculation in *nor* fruit did not appear to induce significant ethylene production as in *rin* and *Cnr* fruit. These results indicate that high levels of ethylene are not required for *B. cinerea* resistance and most likely promote susceptibility. Regardless, the combination of hormone activity and defense gene enrichment suggests that, with the exception of *Cnr* MG-like fruit, resistance or susceptibility in the non-ripening mutants cannot be merely explained by the presence and/or magnitude of immune responses.

### Fruit infections are promoted by a decrease in preformed defenses and an increase in susceptibility factors during ripening

To identify genes that are involved in resistance or susceptibility that change during tomato fruit ripening, we used a differential expression analysis (*P*_adj_<0.05) comparing healthy RR/RR-like to healthy MG/MG-like fruit for each wild-type and mutant line. In wild-type fruit, 6574 genes were significantly down-regulated in RR fruit compared with MG, while 5674 genes were significantly up-regulated (see Supplementary Table S4). We used the susceptibility phenotypes and the transcriptional profiles of the mutant fruit to filter these ripening-associated genes and identify critical preformed defense mechanisms or susceptibility factors. Of the four genotypes, all except *nor* experience an increase in susceptibility in the transition from MG/MG-like to RR/RR-like fruit. Thus, we selected ripening-associated genes that showed the same expression pattern in wild-type, *Cnr*, and/or *rin*, but not *nor*. This filtering resulted in 2893 down-regulated and 2003 up-regulated genes, respectively.

We assumed that effective preformed defenses will decrease during ripening. Thus, the set of filtered down-regulated genes, being those that are highly expressed in healthy MG fruit compared with healthy RR fruit, should contain key genes related to preformed defenses. The filtered down-regulated genes contained 251 defense genes, while up-regulated genes included only 171 defense genes, indicating a net loss of about 80 genes in the transition from MG/MG-like to RR/RR-like susceptible fruit. Furthermore, the 251 defense genes from the filtered down-regulated set were over-represented by functional categories involved in reactive oxygen species (ROS) response and detoxification, proteolysis, and the biosynthesis of secondary metabolites (Table 1). These down-regulated ROS-related genes spanned several subfamilies including thioredoxins, glutaredoxins, glutathione *S*-transferases, and peroxidases. Among the down-regulated proteolytic genes were several subtilisin-like proteases, including *SBT3* (*Solyc01g087850*; Meyer *et al.*, 2016). Lastly, in addition to several genes involved in the methylerythritol 4-phosphate pathway of terpenoid biosynthesis, two copies of the lignin biosynthesis gene *CCoAOMT* (*Solyc01g107910*, *Solyc04g063210*) were also among the filtered down-regulated class, suggesting that cell wall fortification could be inhibited upon infection. These results indicate that ripening involves a loss of multiple defense genes, and that the pre-existing levels of genes involved in ROS regulation, proteolysis, and secondary metabolite biosynthesis may be critical for resistance.

**Table 1.** Defense categories enriched in a subset of significantly down-regulated genes during ripening of healthy tomato fruit

Defense category	Number of genes	Example functions
Cell redox homeostasis (GO:0045454)	24	Thioredoxins, glutaredoxins
Defense response (GO:0006952)	6	MLO-like proteins, Sn-1 proteins
Proteolysis (GO:0006508)	36	Subtilisin-like proteases (SBT2, SBT3)
Response to oxidative stress (GO:0006979)	16	Peroxidases
Flavonoid biosynthesis (sly00941)	5	Caffeoyl-CoA *O*-methyltransferase
Glutathione metabolism (sly00480)	18	Glutathione *S*-transferases
MAPK signaling pathway (sly04016)	17	Protein phosphatase 2C, RBOH proteins
Phosphatidylinositol signaling system (sly04070)	5	Phosphatidylinositol phospholipase C
Plant–pathogen interaction (sly04626)	15	Disease resistance protein RPM1
Terpenoid backbone biosynthesis (sly00900)	8	Geranylgeranyl disphosphate synthase
CAMK	8	Calcium-dependent kinases
RLK	78	Lectin receptor kinases, leucine-rich repeat kinases
ERF	8	ERFA2, ERFC2, ERFC3

The significance cut-off for the enrichments is *P*_adj_<0.05. Full enrichment results for both up-regulated and down-regulated defense genes can be found in Supplementary Table S8.

Finally, we evaluated filtered up-regulated genes that are highly expressed in healthy RR fruit compared with healthy MG fruit, as they may include potential susceptibility factors. Since there is little scientific literature on classes of genes that may constitute susceptibility factors in plants, we focused on the up-regulated genes that were highly expressed in the RR/RR-like fruit of the susceptible genotypes. Such genes may have disproportionate impacts on susceptibility due to their high expression. To identify these genes, we calculated average normalized read count values for each gene across WT, *Cnr*, and *rin* RR/RR-like fruit. The distribution of these values over the filtered up-regulated genes is a notably long-tailed one with a range of 2.43 to 179 649.29 and an average of 1295. We identified genes with abnormally high expression values by selecting outliers (i.e. values above 1.5×the interquartile range) from a log_10_-transformed distribution of the data. This resulted in a list of 16 genes (Table 2). They include several genes previously discovered to be active during tomato fruit ripening, including the flavor volatile biosynthesis gene *ADH2* (*Solyc06g059740*; Speirs *et al.*, 1998), the carotenoid biosynthesis gene *Z-ISO* (*Solyc12g098710*; Fantini *et al.*, 2013), the pectin-degrading enzymes *PG2a* (*Solyc10g080210*; Sheehy *et al.*, 1987) and *PL* (*Solyc03g111690*; Uluisik *et al.*, 2016), and the ethylene receptor *ETR4* (*Solyc06g053710*; Tieman and Klee, 1999), among other genes involved in carbohydrate metabolism.

**Table 2.** Highly expressed genes in susceptible RR/RR-like fruit

Accession	Average RR/ RR-like expression	Name	Ripening function
*Solyc06g059740*	99 772.18	*SlADH2*	Flavor aldehyde biosynthesis
*Solyc08g065610*	64 989.08	*SlVPE3*	Sugar metabolism
*Solyc03g111690*	25 643.87	*SlPL*	Pectin degradation
*Solyc10g080210*	25 044.06	*SlPG2a*	Pectin degradation
*Solyc08g014130*	21 514.72	*SlIPMS2*	Unknown
*Solyc10g076510*	20 051.40	*—*	Unknown
*Solyc07g047800*	19 462.21	*—*	Unknown
*Solyc12g005860*	19 048.01	—	Unknown
*Solyc08g080640*	17 227.90	*SlNP24*	Unknown
*Solyc12g098710*	15 070.45	*SlZ-ISO*	Carotenoid biosynthesis
*Solyc09g009260*	14 572.63	*SlFBA7*	Sugar metabolism
*Solyc10g024420*	14 103.56	—	Unknown

Names and ripening functions were determined via BLAST and literature searches.

**Fig. 5.**
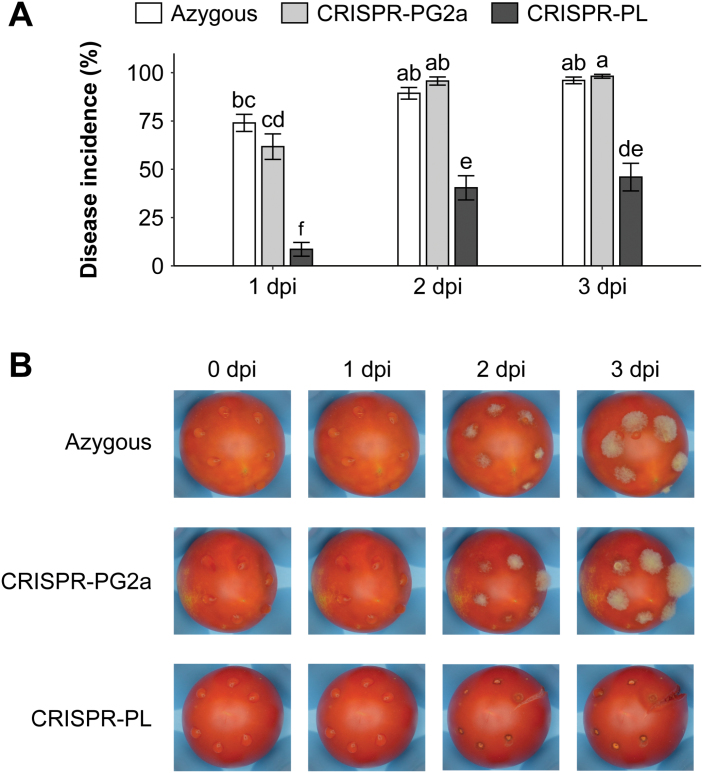
Inoculations of CRISPR lines with *Botrytis cinerea*. (A) Disease incidence measurements at 1, 2, and 3 dpi. Letters represent statistical differences between genotypes (*P*<0.05). (B) Photos of representative inoculated tomatoes from 0 to 3 dpi.

While any of these genes has the potential to impact susceptibility, genes for cell wall-degrading enzymes, such as *PL* and *PG2a*, which facilitate fruit softening during ripening, represent especially good candidates given both the importance of cell wall integrity in defense against fungal pathogens and previous research on RNAi-developed mutants in tomato (Cantu *et al.*, 2008; Yang *et al.*, 2017). To validate the impact of *PG2a* and *PL* expression in wild-type RR fruit on susceptibility to *B. cinerea*, we utilized CRISPR-based mutants in each of these genes (Wang *et al.*, 2019a). RR fruit from these lines are similar in regards to soluble solids content, titratable acidity, and juice pH, but CRISPR-PL fruit are nearly 30% firmer than fruit from the CRISPR-PG2a and azygous WT control lines (see Supplementary Table S2). In conjunction with these firmness differences, RR fruit of the CRISPR-PL line, but not the CRISPR-PG2a line, demonstrated reduced susceptibility to *B. cinerea* compared with the azygous line (Fig. 5). At 3 dpi, disease incidence in the CRISPR-PL fruit was 56% lower than that in azygous fruit. We conclude that the ripening-associated pectate lyase enzyme is a major susceptibility factor for *B. cinerea* infection in tomato fruit.

## Discussion

During ripening, fruit may gradually lose either the ability to activate or the effectiveness of components of the plant immune system, defensive hormone production and signaling, and downstream transcriptional responses. Alternatively, ripening processes such as cell wall breakdown, simple sugar accumulation, changes in pH and secondary metabolite composition, and, in climacteric fruit, increased levels of ethylene may impact the fruit’s capability to resist fungal attack (Prusky *et al.*, 2013; Alkan and Fortes, 2015). The widespread nature of this phenomenon in diverse fruit pathosystems suggests that ripening-associated susceptibility is likely to be mediated by combinations of the above factors.

In tomato, ripening-associated susceptibility has been demonstrated not only for the model necrotrophic pathogen *B. cinerea*, but for other fungal pathogens including *Colletotrichum gloeosporioides* (Alkan *et al.*, 2015), *R. stolonifer*, and *F. acuminatum* (Petrasch *et al.*, 2019). Here, for the first time, we identified specific host responses in both resistant unripe (MG) and susceptible ripe (RR) fruit that are common to multiple pathogens and thus represent core responses to fungal infection. Most prominently, these core responses featured RLKs, WRKY and ERF transcription factors, JA biosynthesis, and chitin catabolism (Fig. 2). Some genes that appear in both the MG core and the RR core responses were previously studied components of plant immunity in tomato, including the JA biosynthesis gene *LoxD* (Yan *et al.*, 2013), the subtilisin-like protease *SBT3* (Meyer *et al.*, 2016), the peroxidase *CEVI-1* (Mayda *et al.*, 2000), and the chitinase *CHI9* (Danhash *et al.*, 1993). Though response to inoculation overlaps somewhat with response to wounding in MG fruit (Supplementary Fig. S1B), transcriptional profiles (Fig. 1B), and ethylene measurements (Supplementary Fig. S2B) indicate that the bulk of inoculation responses are a direct result of fungal attack. This is also evident by the presence of a necrotic ring only in inoculated MG fruit and not in the wounded controls or the inoculated RR fruit, indicating that the unripe fruit is capable of inducing an oxidative burst in response to the pathogen presence (Cantu *et al.*, 2009).

However, most defense genes uncovered were found solely in the RR core response. These included several well-known defense genes that were only expressed in RR fruit, such as *WRKY33* (Liu *et al.*, 2015b), the ERF *PTI5* (He *et al.*, 2001; Gu *et al.*, 2002; Wu *et al.*, 2015), the RLK *TPK1b* (Abuqamar *et al.*, 2008), and the MAP kinase *MPK3* (Kandoth *et al.*, 2007; Stulemeijer *et al.*, 2007; Zhang *et al.*, 2018). While the MG core response did contain some defense genes that were not present in the RR core response (Fig. 3), expression of most of these genes was also identified in the RR response to one or two pathogens. Many of the genes in the MG core response were either functionally similar to other RR core response genes or were expressed at low levels. Thus, the ability to mount an immune response does not appear to be compromised in RR fruit.

If defense responses do not determine the outcome of the interaction in tomato fruit, developmental features associated with ripening of healthy fruit may instead govern susceptibility. The highly complex transcriptional reprogramming during ripening allows for a large number of potential contributors to the increase in susceptibility. Ripening processes in tomato have been studied using non-ripening mutants such as *Cnr*, *rin*, and *nor.* In addition to being phenotypically distinct, these mutants display differential susceptibility patterns when inoculated with fungal pathogens (Fig. 4). Previously, susceptibility to *B. cinerea* in tomato fruit was shown to be dependent on *NOR* but not *RIN*, though the role of *CNR* remained uncharacterized (Cantu *et al.*, 2009). Our results with *B. cinerea* as well as *F. acuminatum* and *R. stolonifer* corroborate the roles of *NOR* and *RIN* while also proposing a role for *CNR* in tomato fruit defense against fungal pathogens. In addition to exhibiting hypersusceptibility to *B. cinerea* in RR-like fruit, *Cnr* MG-like fruit were the only fruit of this stage to exhibit any susceptibility. Unlike *rin* and *nor* fruit, *Cnr* fruit have altered cell wall architecture even in MG-like stages (Eriksson *et al.*, 2004; Ordaz-Ortiz *et al.*, 2009), a feature which may be exploited during fungal infection. Moreover, compared with all other fruit, *Cnr* MG-like fruit were deficient in defense responses against *B. cinerea*. Apart from *Cnr* MG-like fruit, the extent of the immune responses appeared to have little impact on susceptibility, as enriched defense categories were similar across both resistant and susceptible mutant fruit.

We took advantage of the susceptibility differences in the ripening mutants to unravel ripening components that may represent either declining preformed defenses or increasing susceptibility factors. Differential expression analyses carefully filtered based on susceptibility phenotypes revealed that several defense-related genes undergo changes in gene expression during the transition from MG/MG-like to RR/RR-like fruit. Most interestingly, declining preformed defenses appear to be over-represented by gene categories involved in the mediation of ROS levels. Host regulation of ROS levels during early fungal infection is critical for both defense signaling and detoxification of ROS generated by the pathogen (Lehmann *et al.*, 2015; Waszczak *et al.*, 2018), and tomato fruit susceptibility to *B. cinerea* has been shown to be impacted by both of these roles. Improved resistance to *B. cinerea* in the ABA-deficient *sitiens* mutant has been shown to be the result of controlled ROS production, which promotes cell wall fortification (Asselbergh *et al.*, 2007; Curvers *et al.*, 2010), and a similar improved *B. cinerea* resistance is seen in tomato varieties genetically engineered to produce especially high amounts of antioxidant anthocyanins in fruit (Zhang *et al.*, 2015). During ripening, losing control of ROS levels may thus represent the reduction of an important preformed defense.

Some features of ripening have the potential to be either a preformed defense or a susceptibility factor depending on the context. The ethylene burst that accompanies ripening in climacteric fruit is an example. Although ethylene is known for its involvement in defense against necrotrophs (van der Ent and Pieterse, 2012), its induction of the ripening program catalyses downstream events that can be favorable for pathogen infections. Previous research suggests that inhibition of ethylene receptors in MG fruit can either increase or decrease resistance to *B. cinerea* depending on the concentration of inhibitor used (Blanco-Ulate *et al.*, 2013). Thus, ethylene-mediated resistance may be dependent on careful regulation of ethylene levels, and the autocatalytic ethylene biosynthesis that occurs in wild-type fruit ripening may be detrimental. We observed that ethylene production and ethylene-related transcriptional responses were particularly prominent in susceptible fruit, especially *Cnr* MG-like. In addition to ethylene, JA is known to mediate resistance to necrotrophs in plants (Wasternack and Hause, 2013; Pandey *et al.*, 2016). The enrichment of JA biosynthesis genes is seen in the RR core response, as well as the response to *B. cinerea* in all mutant fruit at both stages. Basal levels of JA in healthy fruit are highest in *nor* RR-like fruit, where they are nearly twice as high as levels in wild-type RR fruit. Moreover, *nor* fruit are the only fruit at which JA signaling/response genes are enriched in response to *B. cinerea* infection at both stages. The interplay between ethylene and JA and their impact of ripening-associated susceptibility requires further study.

Other features of ripening can increase susceptibility to fungal disease such as the disassembly of plant cell walls leading to fruit softening. Cell wall polysaccharide remodeling, breakdown, and solubilization in ripening fruit occurs as the result of various cell wall-degrading enzymes, particularly those that act on pectin (Brummell, 2006). The cell wall represents an important physical barrier to pathogen attack in plants (Malinovsky *et al.*, 2014; Blanco-Ulate *et al.*, 2016a), and cell wall integrity and fortification improves tomato fruit resistance to *B. cinerea* infection (Cantu *et al.*, 2008; Curvers *et al.*, 2010). The enzymes PL and PG2a feature prominently in tomato fruit ripening and softening (Uluisik *et al.*, 2016; Yang *et al.*, 2017; Wang *et al.*, 2019a) and accumulate in RR/RR-like fruit of susceptible genotypes. However, these enzymes do not have equal impact on fruit softening, as CRISPR-based mutants in *PL*, but not *PG2a*, result in a reduced rate of softening in RR fruit (Wang *et al.*, 2019a). This differential impact on firmness is mirrored in the effect on susceptibility to *B. cinerea*, as the firmer CRISPR-PL mutant was less susceptible than both the CRISPR-PG2a mutant and the azygous control (Fig. 5). Though RR fruit of the CRISPR-PG2a mutant did not exhibit increased *B. cinerea* resistance, PG2a may still contribute to susceptibility, as RNAi-mediated knockdown of *PG2a* together with the expansin gene *Exp1* increases *B. cinerea* resistance while knockdown of either gene alone does not (Cantu *et al.*, 2008). Here we showed that the PL enzyme is a substantial susceptibility factor in tomato fruit, and targeting this enzyme for breeding purposes may improve fungal resistance in addition to lengthening shelf life by slowing the softening process.

**Fig. 6.**
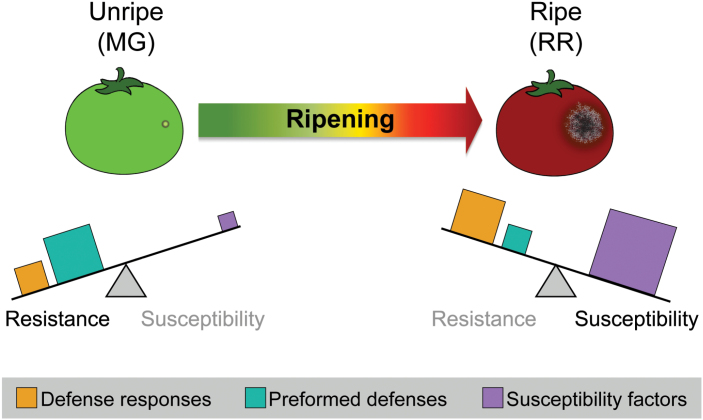
Model of contributing factors to ripening-associated susceptibility in tomato fruit. Sizes of squares indicate the relative magnitude of that feature in fruit of that stage. The balance between contributing components determines the ultimate outcome of the infection. MG, mature green; RR, red ripe.

Susceptibility and resistance to necrotrophic pathogens is ultimately a complex, multigenic trait in plants. The use of transcriptomic datasets to facilitate a systems-level approach of such pathosystems has increased in recent years (Alkan *et al.*, 2015; Kovalchuk *et al.*, 2019; Petrasch *et al.*, 2019; Zhang *et al.*, 2019) and has led to novel insights in both host and pathogen features that impact the outcome of such interactions. Moreover, the additional layer of an enormous developmental change such as ripening only further increases the need for these approaches. We have demonstrated how such an approach can yield critical information on both fruit infection response and broad ripening-associated changes that increase susceptibility, and additionally provide insights into single genes with a disparate impact on susceptibility. From our results, we believe that ripening-associated susceptibility is best explained by a dominant role of susceptibility factors that increase during ripening, which, coupled with a modest loss of preformed defenses, outweighs the efforts of the immune response in ripe fruit (Fig. 6). Overall, our results have tremendous utility for guiding future study of fruit–pathogen interactions in addition to providing breeders with information on potentially useful genes for targeting in the hopes of ultimately reducing postharvest losses in tomatoes and other fruit crops.

## Supplementary data

The following supplementary data are available at *JXB* online.

Fig. S1. Pathogen measurements and wound responses.

Fig. S2. Defense responses and ethylene levels in wild-type and mutant fruit.

Table S1. Physiological measurements of fruit from wild type (cv. Ailsa Craig) and the isogenic non-ripening mutants *Cnr*, *nor*, and *rin*.

Table S2. Physiological measurements of red ripe fruit from azygous, CRISPR-PL, and CRISPR-PG2a lines.

Table S3. Summaries of RNA-sequencing read mapping to tomato and pathogen transcriptomes.

Table S4. Differential expression output from DESeq2 (Love *et al.*, 2014) with functional annotations.

Table S5. Common and unique DEGs for each pathogen at 1 DPI in MG and RR fruit.

Table S6. Core RR response defense genes not expressed at equal or greater levels than MG in infected fruit.

Table S7. Jasmonic acid measurements from healthy wild-type and non-ripening mutant fruit.

Table S8. Enrichment of defense genes in filtered up-regulated/down-regulated ripening genes.

eraa601_suppl_Supplementary_Figures_S1-S2

eraa601_suppl_Supplementary_Table_S1-S8

eraa601_suppl_Supplementary_Table_S4

eraa601_suppl_Supplementary_Table_S5

## Data Availability

The datasets for this study have been deposited in the Gene Expression Omnibus (GEO) database under the accession GSE148217.
