# Supplementary material for: Host susceptibility factors render ripe tomato fruit vulnerable to fungal disease despite active immune responses
Source: J Exp Bot. 2021 Jan 19;72(7):2696–709. doi: 10.1093/jxb/eraa601 (PMC8006553; doi:10.1093/jxb/eraa601)
Supplement: eraa601_suppl_Supplementary_Figures_S1-S2 [file eraa601_suppl_supplementary_figures_s1-s2.pdf]

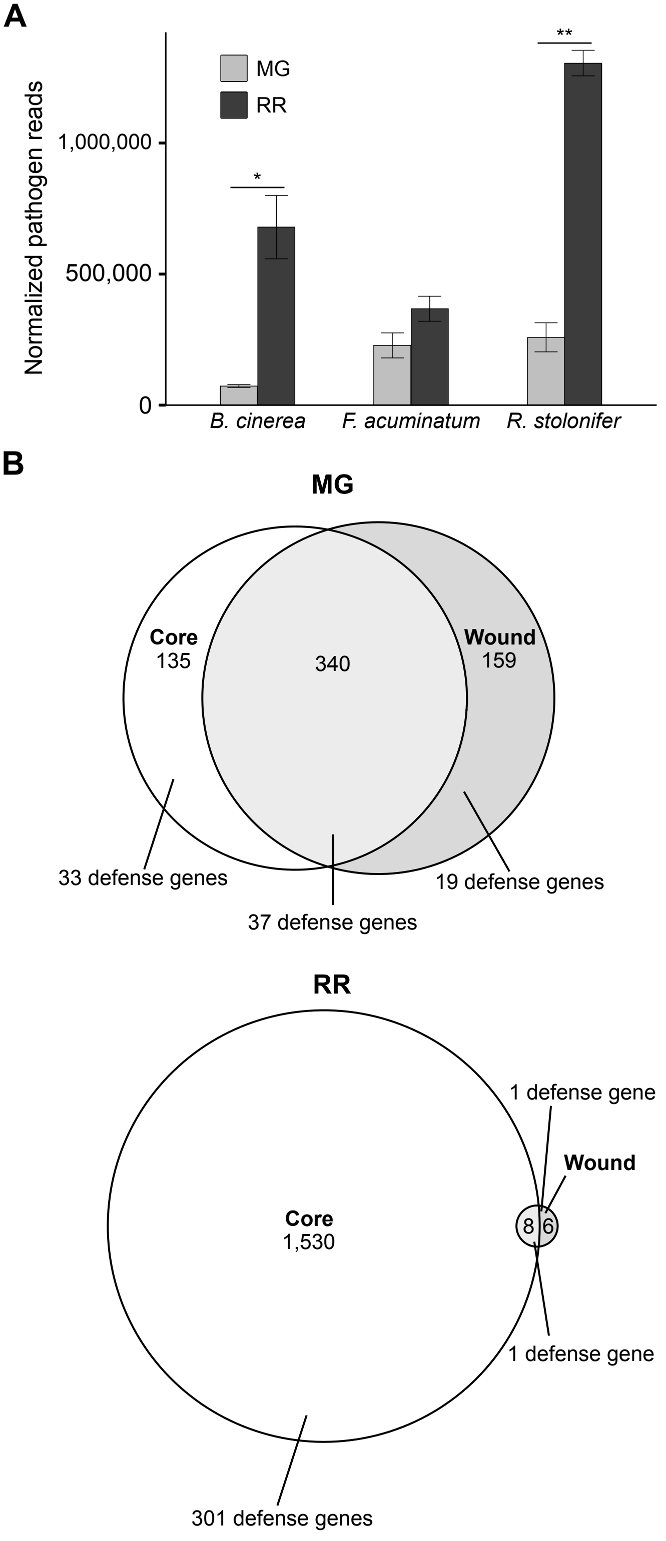

Supplemental Fig. S1. Pathogen measurements and wound responses. (A) Normalized pathogen reads from fruit inoculated with the corresponding pathogen at mature green (MG) and red ripe (RR) stages. Asterisks indicate level of significance of t-test between MG and RR stages for each pathogen. * = $P < 0.05$, ** = $P < 0.01$. **(B)** Comparison of MG and RR core responses with genes upregulated during wounding response in fruit of the same stage. Number of defense genes are indicated for the subsets.

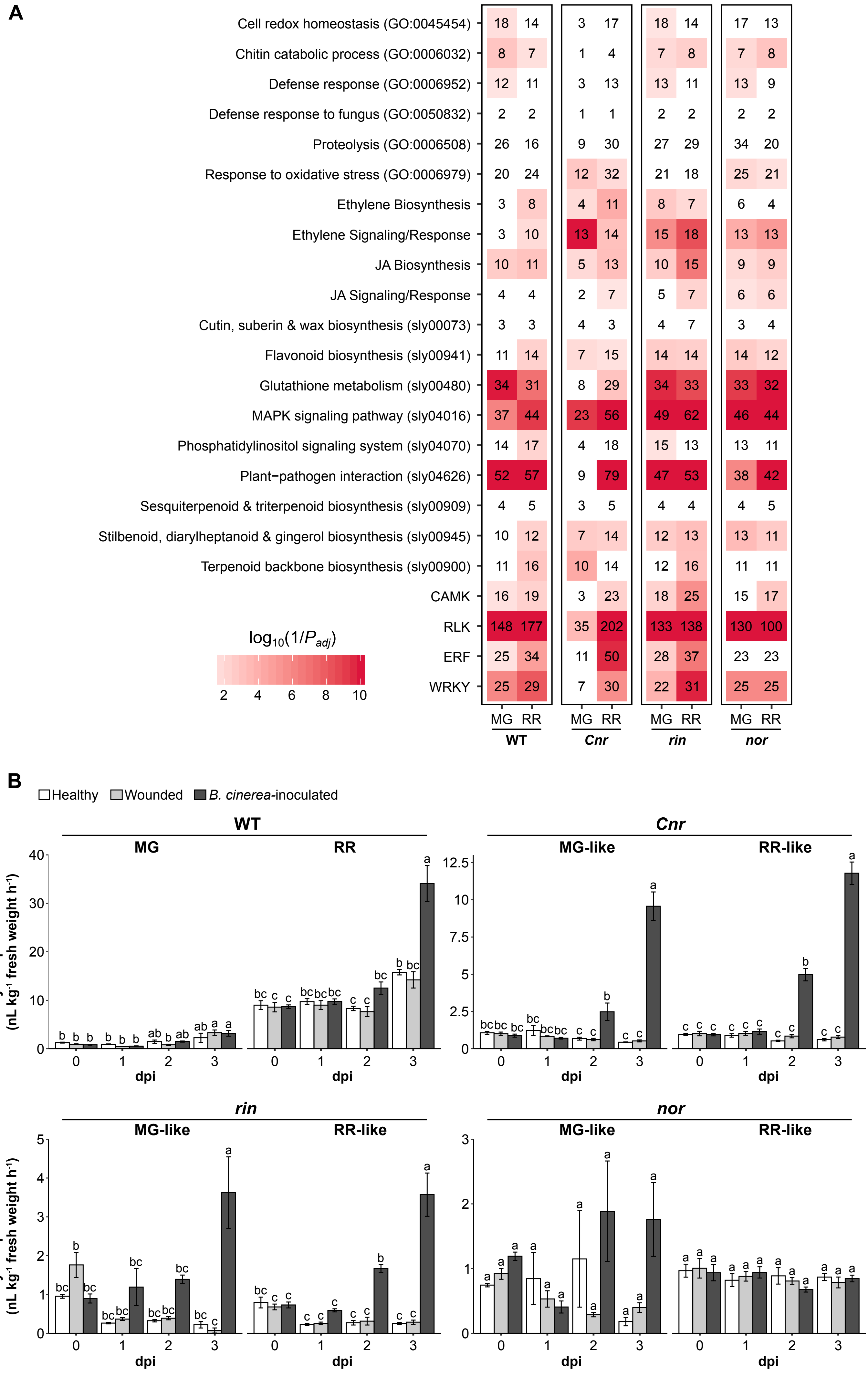

Supplemental Fig. S2. Defense responses and ethylene levels in wild-type and mutant fruit. (A) Enrichments of various defense-related classes in the MG/MG-like and RR/RR-like responses. The scale is the $\log_{10}(1/P_{adj})$. Values greater than 10 were converted to 10 for scaling purposes. **(B)** Levels of ethylene production in healthy, wounded, and *B. cinerea*-inoculated MG/MG-like and RR/RR-like fruit from 0 to 3 days post-inoculation (dpi). Letters represent statistical differences between treatments and time points for each genotype and ripening stage ($P < 0.05$). MG = mature green, RR = red ripe.
